# A fine-grained Chinese word segmentation and part-of-speech tagging corpus for clinical text

**DOI:** 10.1186/s12911-019-0770-7

**Published:** 2019-04-09

**Authors:** Ying Xiong, Zhongmin Wang, Dehuan Jiang, Xiaolong Wang, Qingcai Chen, Hua Xu, Jun Yan, Buzhou Tang

**Affiliations:** 1 Department of Computer Science, Harbin Institute of Technology Shenzhen Graduate School, Shenzhen, China; 2 Department of Information Technology, The First Affiliated Hospital of Nanjing Medical University, Nanjing, China; 3 School of Biomedical Informatics, The University of Texas Health Science Center at Houston, Houston, TX USA; 4 Yidu Cloud (Beijing) Technology Co.,Ltd, Beijing, China

**Keywords:** Fine-grained Chinese word segmentation, Part-of-speech tagging, Clinical named entity recognition

## Abstract

**Background:**

Chinese word segmentation (CWS) and part-of-speech (POS) tagging are two fundamental tasks of Chinese text processing. They are usually preliminary steps for lots of Chinese natural language processing (NLP) tasks. There have been a large number of studies on CWS and POS tagging in various domains, however, few studies have been proposed for CWS and POS tagging in the clinical domain as it is not easy to determine granularity of words.

**Methods:**

In this paper, we investigated CWS and POS tagging for Chinese clinical text at a fine-granularity level, and manually annotated a corpus. On the corpus, we compared two state-of-the-art methods, i.e., conditional random fields (CRF) and bidirectional long short-term memory (BiLSTM) with a CRF layer. In order to validate the plausibility of the fine-grained annotation, we further investigated the effect of CWS and POS tagging on Chinese clinical named entity recognition (NER) on another independent corpus.

**Results:**

When only CWS was considered, CRF achieved higher precision, recall and F-measure than BiLSTM-CRF. When both CWS and POS tagging were considered, CRF also gained an advantage over BiLSTM. CRF outperformed BiLSTM-CRF by 0.14% in F-measure on CWS and by 0.34% in F-measure on POS tagging. The CWS information brought a greatest improvement of 0.34% in F-measure, while the CWS&POS information brought a greatest improvement of 0.74% in F-measure.

**Conclusions:**

Our proposed fine-grained CWS and POS tagging corpus is reliable and meaningful as the output of the CWS and POS tagging systems developed on this corpus improved the performance of a Chinese clinical NER system on another independent corpus.

## Background

Chinese word segmentation (CWS) and part-of-speech (POS) tagging are two fundamental tasks of Chinese text processing, which are preliminary steps of Chinese natural language processing (NLP) tasks, such as named entity recognition (NER), information retrieval, machine translation, etc. The two tasks have always attracted plenty of attention as CWS is still challenging especially in specific domains. In the clinical domain, there have been few studies proposed for CWS and POS tagging because it is difficult to determine granularity of words, which is different from other domains such as newswire. For example, “自觉” is a word that usually means “conscientiously” in the newswire domain, but are two words “自” (self) and “觉” (feel) that mean “feels ... by himself/herself” in the clinical domain. “上下颚” (the upper and lower jaws), which depicts two body parts “上颚” (the upper jaw) and “下颚” (the lower jaw), should be split into three words “上” (upper), “下” (lower) and “颚” (jaw) as only in this way is it possible to form the two body parts “上颚” (the upper jaw) and “下颚” (the lower jaw) by combining “上” (upper) and “下” (lower) with “颚” (jaw), respectively, which are very important for subsequent tasks such as clinical named entity recognition and normalization.

In order to make sure that CWS and POS tagging for clinical text are consistent with subsequent clinical NLP tasks, we investigated the two fundamental tasks at a fine-grained level comprehensively, and manually annotated a benchmark corpus composed of 1800 clinical notes from a tier 3A hospital of China. On this corpus, we first compared two state-of-the-art methods, i.e., conditional random fields (CRF) and bidirectional long short-term memory (BiLSTM) with a CRF layer (called BiLSTM-CRF), and then further investigated the effect of CWS and POS tagging on Chinese clinical named entity recognition (NER) on another independent benchmark corpus, that is the corpus for task 1 of the CCKS (China conference on knowledge graph and semantic computing) challenge in 2017. Both CRF and BiLSTM-CRF achieved state-of-the-art performance in CWS and POS tagging, and the output of the CWS and POS tagging systems improved the performance of a BiLSTM-CRF-based Chinese clinical NER system when they were simply integrated into the system as a part of input. The experimental results indicate that our proposed fine-grained CWS and POS tagging corpus is reliable and meaningful.

### Related work

CWS and POS tagging have been widely being investigated for a long time as they are two fundamental tasks in NLP. Both of them are regularly recognized as sequence labeling problem, and a large number of machine learning methods have been proposed for them, including machine learning methods relying on manually-crafted features such as maximum entropy Markov model [1] conditional random fields [2] structural support vector machines [3] etc., and deep learning methods that do not need manually feature engineering such as BiLSTM-CRF [4], CNN (convolutional neural networks)-CRF [5] and their variants [6–8]. The deep learning methods usually shows better performance than the machine learning methods relying on manually-crafted features. Most of these studies focused on algorithms rather than other aspects such as domain transfer [9, 10] and multiple labeling criteria [11]. However, in recent years, application needs of NLP in specific domains such as clinic, finance, law, etc., have become more and more. A few researchers began to investigate domain-specific NLP techniques.

In the Chinese clinical domain, there have been a small number of studies on NLP tasks, including CWS, POS tagging, latent syntactic analysis, parsing, de-identification, NER, temporal information extraction, etc. In the case of CWS and POS tagging, the existing work was mainly carried out from a linguistics perspective, and might not be suitable for actual applications. Therefore, we investigated CWS and POS tagging for Chinese clinical text at a fine-grained level according to application needs.

## Materials and methods

**Fig. 1:**
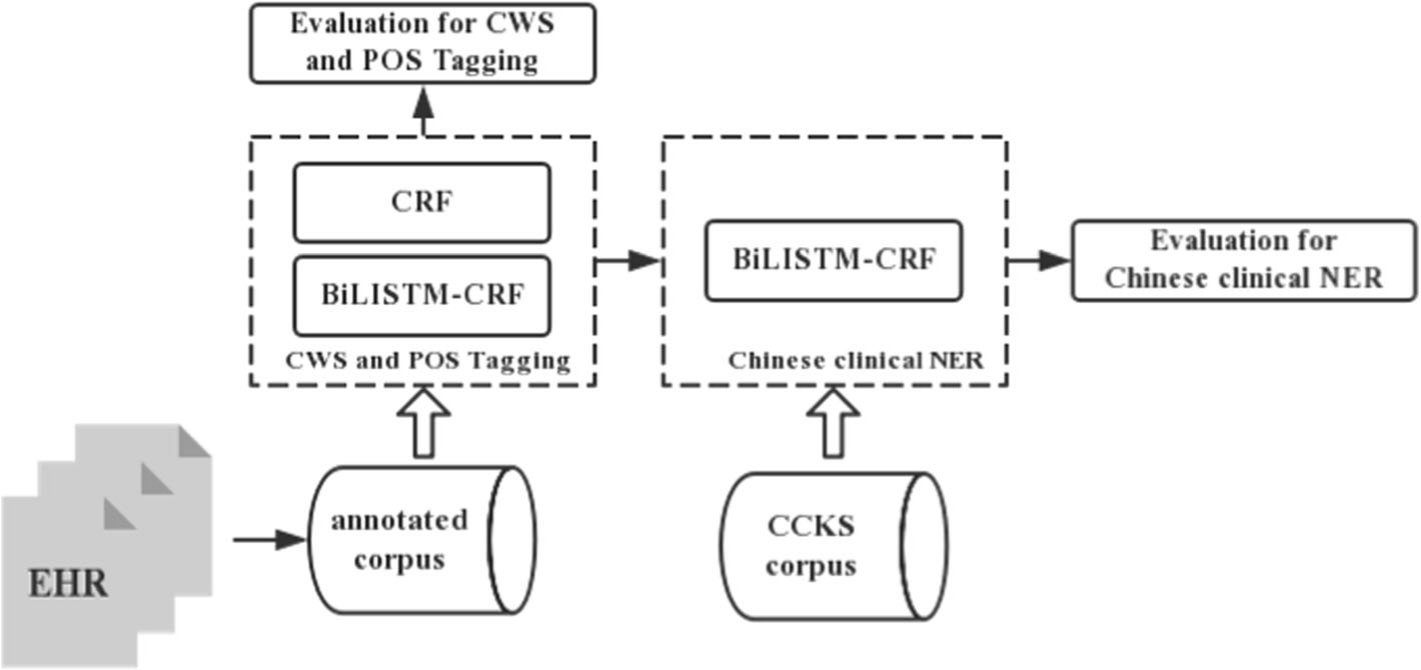
Workflow of our study

As shown in Fig. 1, we first annotated a fine-grained corpus, then compared two state-of-the-art machine learning methods, that is CRF, a machine learning method based on manually-crafted features, and BiLSTM-CRF, a deep learning method that does not rely on manaully-crafted features, for CWS and POS tagging for clinical text on the corpus, and finally investigated the effect of CWS and POS tagging on Chinese clinical NER on another benchmark dataset (CCKS2017) by comparing the performance change of a BiLSTM-CRF-based Chinese clinical NER system before or after adding CWS and POS tagging information as a part of input.

### Dataset and annotation

The corpus was collected from the electronic health record (EHR) system of a tier 3A hospital in China. After excluding fragmentary clinical notes, a total of 1800 clinical notes, including 1000 admission notes and 800 discharge summaries, were selected for this study. Before annotation, all personal health identifiers were manually removed from the clinical notes, and a fine-grained annotation guideline was developed by three medical experts and four master students in NLP. We followed the processing specification of national 973 plan of China for contemporary Chinese word segmentation and POS tagging, and further split words that may cause inconsistencies in subsequent tasks into smaller ones. The cases we mainly concerned are: (1) nouns of locality split away from body parts when they are pre-modifiers of body parts. For example, “左上肢/NN(the left upper arm)” is segmented into “左/JJ(left) 上/JJ(upper) 肢/NN(arm)” consistent with “上下颚” (the upper and lower jaws) mentioned above, where NN represents noun, and JJ represents adjective. However, when noun(s) of locality is (are) a post-modifier(s) of body parts and the noun(s) together with body parts form a noun, the nouns do not split away from body parts. For example, “腋下/NN (underarms)”; (2) modal verbs and action words are separated. For example, “能动(be able to move)” is segmented into “能/VV(be able to)” and “动/VV(move)”, where “VV” represents verb; (3) labtests and procedures that consist of a verb and a noun, which can be replaced by other similar nouns, are split into a verb and a noun. For example, “禁水(abstain from drink)” is split into “禁/VV(abstain from)” and “水/NN(drink)” as “水/NN(drink)” can be replaced by “食(food)”. Only in this way “禁水(abstain from drink)” and “禁食(abstain from food)” are possible to be extracted from “禁水食(abstain from drink and food)” subsequently; (4) two or more body parts appear in parallel are separated. For example, “耳鼻喉(ear, nose and throat)” is segmented into “耳/NN(ear)”, “鼻/NN(nose)” and “喉/NN(throat)”, “手足(hand and foot)” is segmented into “手/NN(hand)” and “足/NN(foot)”, “肝胆(hepatobiliary and pancreatic)” is segmented into “肝/NN (hepatobiliary” and “胆(pancreatic)”, etc.

After annotation, we obtained 198,797 words with average length of 1.5, among which, the numbers of words with length of 1, 2, 3 and more than 3 are 108,250, 64,823, 10,056, and 15,707, respectively. The annotated corpus were randomly divided into three parts: four-fifths of clinical notes (1440) are used as a training set, tenth (180) of clinical notes as a development set, and the remaindering tenth (180) of clinical notes as a test set. Table 1 lists the statistics of our corpus. In order to ensure consistency, we conducted multiple rounds of check and labeling of the corpus. The inter-annotation agreements using kappa statistics [12] on CWS and POS tagging in the last two rounds were 0.9816 and 0.9810, respectively, which implied that the annotation was reliable.

**Table 1:** Statistics of our fine-grained Chinese word segmentation and part-of-speech tagging Corpus for clinical text

Dataset	Notes	Sentences	words
Training	1440	6867	158,035
valid	180	813	19,290
test	180	857	21,472
total	1800	8537	198,797

The CCKS2017 is the only one corpus publicly released currently for Chinese clinical NER, which contains a training dataset of 300 clinical records and a test dataset of 100 clinical records. In this corpus, five types of clinical entities (body, disease, symptom, test and treatment) are manually annotated. The number of entities in each type is listed in Table 2. In our study, we randomly separated 100 clinical records out from the training set as an independent development set.

**Table 2:** Statistics of entities on different categories of CCKS

Dataset	Body	Disease	Symptom	Test	Treatment	Total
Train	10,719	722	7831	9546	1048	29,866
Test	3021	553	2311	3143	465	9493

### Tagging schema

In our study, CWS and POS tagging were regarded as a sequence labeling problem. All words in Chinese were represented by the tagging schema “BMES”, where ‘B’, ‘M’, and ‘E’ represent that the current Chinese character is at the beginning, middle and end of a word, ‘S’ represents the current Chinese character is a word with length 1. We considered the following two cases for CWS and POS tagging: (1) CWS only; and (2) CWS and POS tagging simultaneously. Table 3 gives some examples of CWS and POS tagging representation where ‘CD’ represents classifier and ‘M’ represents unit.

**Table 3:** Examples of CWS and POS tagging representation using tagging schema “BMES”

Word segmentation	BMES tags
左眼视力下降数年 (Left eye’s vision have declined for several years) 查血常规 (do complete blood count test)	左/S 眼/S 视/B 力/E 下/B 降/E 数/S 年/S (左-left, 眼-eye, 视力-vision, 下降-decline, 数-several, 年-year) 查/S 血/B 常/M 规/E (查-do ... test, 血常规-complete blood count)
Word segmentation and POS tagging	BMES tags
左眼视力下降数年 (Left eye’s vision have declined for several years)	左/S-JJ 眼/S-NN 视/B-NN 力/E-NN 下/B-VV 降/E-VV 数/S-CD 年/S-M (左-left, 眼-eye, 视力-vision, 下降-decline, 数-several, 年-year)
查血常规 (do complete blood count test)	查/S-VV 血/B-NN 常/M-NN 规/E-NN (查-do ... test, 血常规-complete blood count)

### Experimental settings

We adopted CRF++ (https://taku910.github.io/crfpp) as an implementation of CRF, and Information Extraction (https://github.com/crownpku/Information-Extraction-Chinese) as an implementation of BiLSTM-CRF. The features used by CRF included unigrams, bigrams and trigrams in a window of [− 2, 2] around a work of interest. In the BiLSTM-CRF model, 50-dimensional embeddings, 10-dimensional embeddings.

and 20-dimensional embeddings were used to represent Chinese characters, CWS tag.

of Chinese characters and CWS&POS tag of Character characters, respectively. We followed Hu et al.’s [13] way to initialize the embeddings of Chinese characters, and.

initialized other embeddings randomly. The other hyperparameters were set as follows:learning rate-0.001, batch size-20, dropout rate-0.5 and optimizer-adam. All parameters were optimized on the development set, and all models were evaluated by precision (P), recall (R) and F-measure (F).

## Results

The performance of CRF and BiLSTM-CRF on CWS and POS tagging for clinical text on our corpus is shown in Table 4. When only CWS was considered, CRF achieved higher precision, recall and F-measure than BiLSTM-CRF. The difference was 0.33% in F-measure. When both CWS and POS tagging were considered, CRF outperformed BiLSTM-CRF by 0.14% in F-measure on CWS and by 0.34% in F-measure on POS tagging. In the case of CWS, when considering CWS and POS tagging simultaneously, each machine learning method achieved a little bit higher F-measure than it considered CWS only.

**Table 4:** Performance of CRF and BiLSTM-CRF on CWS and POS tagging for clinical text on our corpus

Task	Method		P(%)	R(%)	F(%)
CWS	CRF		96.75	97.14	96.94
	BiLSTM-CRF		96.56	96.66	96.61
CWS&POS tagging	CWS	CRF	97.18	96.73	96.95
		BiLSTM-CRF	96.86	96.76	96.81
	POS tagging	CRF	95.34	94.89	95.11
		BiLSTM-CRF	94.81	94.72	94.77

Moreover, we investigated the effect of fine-grained CWS and POS tagging on NER for clinical text. The performance of BiLSTM-CRF using and without using CWS and POS information was shown in Table 5, where “baseline” was the system that only used Chinese character embeddings as input. When CWS or CWS&POS information was added, the performance of BiLSTM-CRF-based NER system was improved, and.

**Table 5:** Effect of fine-grained CWS and POS tagging on NER for clinical text

System	P(%)	R(%)	F(%)
Baseline	88.83	88.74	88.79
+ CWS (CRF)	89.28	88.79	89.13
+ CWS&POS (CRF)	90.90	88.20	89.53
+ CWS (BiLSTM-CRF)	89.31	88.75	89.03
+ CWS&POS (BiLSTM-CRF)	89.65	88.63	89.14

CWS&POS information brought greater improvement than CWS information only in F-measure. The better the performance of the CWS or CWS&POS tagging system (i.e., CRF) was, the higher the F-measure of the NER system was. The greatest improvement in F-measure was 0.74%, obtained from the CWS&POS tagging system based on CRF.

## Discussion

Compared with CWS and POS tagging systems in the newswire domain, our fine-grained CWS and POS tagging system is competitive with a highest F-measure of 96.94% on CWS and that of 95.11% on POS tagging. Unlike in the newswire domain, CRF outperforms BiLSTM-CRF in the Chinese clinical domain. The reason may lie in the following two aspects: 1) most sentences in Chinese clinical notes are not complete sentences in linguistic grammar, in which Chinese characters are locally related; 2) at the fine-grained level, most of words are shorter than 2 Chinese characters, indicating that long dependencies of Chinese characters are not needed for CWS in clinical text. To validate our assumption, we extended the size of the context window for feature extraction in CRF from [− 2, 2] to [− 3, 3], and the performance of CRF dropped.

The reason why fine-grained CWS or CWS&POS information improved the performance of the BiLSTM-CRF-based NER system as shown in Table 5 is that some body parts embedded in some general words can be correctly recognized after CWS or CWS&POS information was added, such as “舌(tongue)” in “伸舌(stick out your tongue)” fact, in the CCKS2017 NER corpus, the cases of many entities sharing some words are not considered when annotation, the effect of fine-grained CWS or CWS&POS information is limited. In the future work, we will investigated the effect of fine-grained CWS or CWS&POS information on more general Chinese clinical NER which considers both continuous and discontinuous entities.

Although the proposed fine-grained CWS and POS tagging system achieved competitive performance, there also existed some errors. For example, the ‘+’ in ‘行ERC+ESC+取石术(under ERC, ESC and EST)’ was recognized as ‘positive’ which is ‘+/M-NN’, however, it is a conjunction. ‘高血压病史(history of hypertension)’ was mistakenly segmented into ‘高/B-NN 血/M-NN 压/M-NN 病/E-NN(hypertension) 史/S-NN(history)’, because there were many ‘糖尿病史(history of diabetes mellitus)’ and they were segmented into ‘糖/B-NN 尿/M-NN 病/E-NN(diabetes mellitus) 史/S-NN(history)’ . In the case of the former error, we may need to understand what ERC and ESC stand for by introducing an extra knowledge base or dictionary that interprets these abbreviations, which is one case of our future work. The latter error may not affect some subsequent tasks such as normalization. We will investigated whether this type of errors affect normalization in the future.

It should be stated that as we split one third of notes out from the training set used for system development when conducting experiments on CCKS2017, our BiLSTM-CRF-based system did not achieve as good performance as [13].

## Conclusion

In this study, to investigate CWS and POS tagging for clinical text at a fine-grained level, we annotated a corpus of 1800 clinical notes, applied CRF and BiLSTM-CRF on this corpus, and achieved competitive performance. Furthermore, we tested the effect of CWS and POS information on Chinese clinical NER. Experimental results on a benchmark dataset show that fine-grained CWS and POS tagging make a great contribution to Chinese clinical NER, and have potential for other clinical application tasks.
